# Metastasectomy as optimal treatment for late relapsing solitary brain metastasis from testicular germ cell tumor: a case report

**DOI:** 10.1186/1756-0500-7-865

**Published:** 2014-12-02

**Authors:** Keitaro Iida, Taku Naiki, Noriyasu Kawai, Ryosuke Ando, Toshiki Etani, Keiichi Tozawa, Kenjiro Kohri

**Affiliations:** Department of Nephro-urology, Nagoya City University Graduate School of Medical Sciences, Kawasumi 1, Mizuho-cho, Mizuho-ku, 467-8601 Nagoya, Japan

**Keywords:** Late relapse, Testicular germ cell tumor, Yolk sac tumor, Brain metastasis, Surgery

## Abstract

**Background:**

Management of late relapse of a testicular germ cell tumor is difficult because few cases have been reported and the tumors are intractable to chemotherapy. Here we present a case with a single brain metastasis from late relapse of a testicular germ cell tumor. This is the first report of a brain metastasis that was treated successfully only by surgery.

**Case presentation:**

A 19-year-old Japanese man presented with breathing difficulties and left testis enlargement and he was diagnosed with a yolk sac tumor following a left orchiectomy. At the time of diagnosis, multiple lung metastases were apparent on computed tomography, and serum alpha-fetoprotein level was elevated to 10,245 ng/ml. The patient received three postoperative courses of bleomycin, etoposide and cisplatin and etoposide and cisplatin respectively and a complete response was obtained. Four years after surgery, the patient was admitted to the hospital due to a sudden seizure. High alpha-fetoprotein levels (539 ng/ml) were evident and magnetic resonance imaging suggested a 45-mm single brain tumor in the right parietal lobe, for which surgery was performed. The pathological diagnosis was yolk sac tumor. The alpha-fetoprotein level remained normal at 2 months after operation. There was no recurrence 24 months post-operation.

**Conclusion:**

Chemoresistance and late neurotoxicity are concerns in treating brain metastasis with chemotherapy or cerebral radiotherapy. Surgery is believed to be the optimal treatment choice if the size of the brain metastasis is larger than 35-mm and the late relapse area is surgically accessible.

## Background

Late relapses (LRs) of a testicular germ cell tumors (GCT) is defined as a recurrence after complete response to treatment with a subsequent disease-free interval of at least of 2 years [1]. LRs of testicular GCT are rare, with an incidence rate of 3- 4% [2]. The most common sites of LR are the retroperitoneum (47%), lungs (25%) and mediastinum (10%) [1]. LRs also appear in cervical nodes (8%) and the liver (6%) [1]. To date, only a few cases of brain metastases of LR of testicular GCTs have been reported. Hence, it is quite difficult to decide on the choice of treatment.

Herein, our experience in a case with a single brain metastasis from a LR of a testicular GCT is described. The patient was successfully treated with surgery alone. This paper describes the characteristics and treatment of LRs of testicular GCTs including brain metastases.

## Case presentation

**Figure 1:**
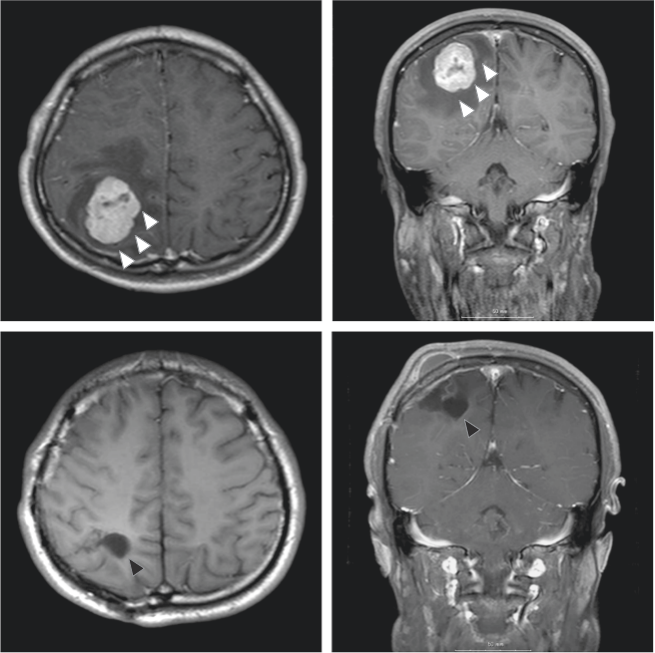
**Magnetic resonance imaging of the brain metastasis.** Above, representative axial and coronal T1-weighed gadolinium-enhanced Magnetic resonance imaging (MRI) slices of the brain at the time of seizure. White arrow: brain tumor. The images reveal a 45-mm mass on the right side of the brain. Below, axial and coronal T1-weighed gadolinium-enhanced MRI slices, taken 6 months post-operation. Black arrow: post-treatment change after operation.

**Figure 2:**
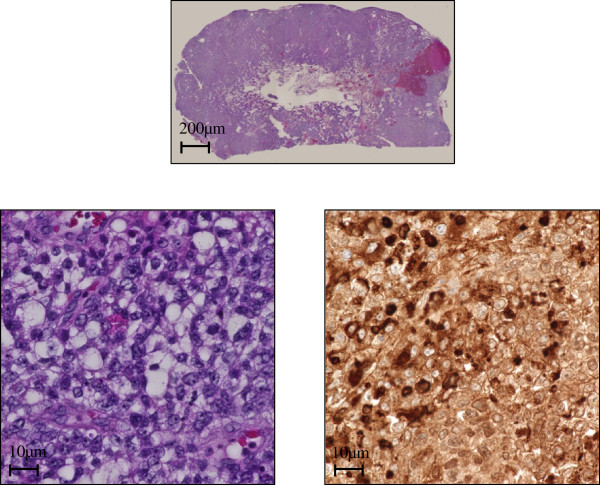
**Hematoxylin and eosin staining of brain metastasis.** Above, low magnification image. Below, high magnifications of brain tumor. Hematoxylin and eosin staining and positive immune-staining for alpha-fetoprotein. The final pathological diagnosis was a yolk sac tumor.

A 19-year-old Japanese man consulted a family physician due to breathing difficulties. The left testis was enlarged to 15 cm in diameter, and multiple lung shadows were observed on a chest X-ray. The patient was then referred to our institution due to suspected lung metastases of testicular cancer. Left orchiectomy was performed and he was diagnosed with a yolk sac tumor (YST). At the time of diagnosis, multiple lung metastases were apparent on computed tomography (CT). Postorchiectomy serum levels of tumor markers were examined, with an elevated alpha-fetoprotein (AFP) level of 10,245 ng/ml, but otherwise normal with lactate dehydrogenase (LDH) level of 151 U/l and human chorionic gonadotrophin (HCG) level of 0.4 IU/L. Elevated AFP level indicated that he fulfilled the criteria of poor prognosis according to the International Germ Cell Consensus Classification [3]. The patient received three postoperative courses of bleomycin, etoposide and cisplatin (BEP) and etoposide and cisplatin (EP) respectively and a complete response was obtained. There was no recurrence post-operatively during follow-ups at 3–6 months intervals, according to serum marker levels and CT imaging from the chest to pelvis. Four years after surgery, the patient was transferred to the emergency department due to a sudden seizure. High AFP levels (539 ng/ml) were evident, although other markers were normal. Magnetic resonance imaging (MRI) suggested a 45-mm single brain tumor in the right parietal lobe, and no other tumor was evident, including the contralateral testis (Figure 1). Surgery was performed on the single brain metastasis. The pathological diagnosis was YST (Figure 2). The AFP level remained normal at 2 months after surgery. The patient has not had neurological disability. Moreover, there was no recurrence 24 months after surgery.

## Conclusions

LR can be due to a second primary tumor (either a metastasis from a second testicular neoplasm or an extragonadal primary tumor), a delayed transformation of a metastatic teratoma into a malignant neoplasm, or persistence of a microscopic viable cancer with slow-growing tendencies [4].

Various histologies have been reported for LR: GCT (66%), pure teratoma (17- 22%), sarcoma (6%), and adenocarcinoma (6%) [1, 2, 5]. Sixty percent of patients present with elements of teratoma [5]. Of those patients with LR of GCT, 44.6% are diagnosed with YSTs, which comprise the most common GCT subtype [2]. A report by Michael et al. included 91 patients with LR of GCT, of whom 43 had YSTs [5]. Of those 43 patients with LR of GCT, only 12 patients were diagnosed with YST as the primary neoplasm. Furthermore, the disease-free rates for patients with LR during long-term follow-up were 74% for those with LR consisting only of teratoma versus, 37% for those with LR consisting of YST.

Testicular GCT responds well to chemotherapy, even with brain metastases, but chemotherapy for LR has been unsuccessful in many patients [5]. According to a study by Baniel, 17 of 65 (26%) patients who received chemotherapy as treatment for LRs of testicular GCTs experienced complete responses. However, only 2 (3%) patients who received chemotherapy alone were continuously disease-free. Of note, these 2 patients had not previously received cisplatin-based chemotherapy, suggesting that LR is associated with chemoresistance [1, 5]. While chemotherapy might be effective for chemotherapy-naive patients, it does not work for most patients with LR if they previously received chemotherapy to treat the primary tumor. Consequently, a surgical approach is the most appropriate treatment choice. George et al. reported that 49 of 83 patients (59%) presented with no evidence of disease after surgery for LR [6]. Twenty-one of the 49 patients (43%) who responded successfully to the primary surgery remained continuously disease-free, while 28 of the 49 patients (57%) experienced one or more subsequent relapses.

The 5-year cause-specific survival for all cases of brain metastasis from testicular GCT ranges from 35.9% to -53% [7–9]. The 5-year cause- specific survival for cases of brain metastasis at initial diagnosis was 42.9%. In contrast, the 5-year cause-specific survival for cases of brain metastasis after or during cisplatin-based chemotherapy was even worse, 12% to -29.6%, indicating that brain metastases in the latter group were chemotherapy-resistant [7–9]. In such cases, cerebral radiotherapy or surgery might provide important benefits.

Cerebral radiotherapy has been demonstrated as a treatment for brain metastases of testicular GCT, as these tumors possess a certain level of radiosensitivity. Stereotactic radiotherapy (SRT) techniques, such as GammaKnife or CyberKnife have been used to treat brain tumors. In general, for brain metastasis, the indication for SRT includes tumors with a median diameter less than 3.5 cm and up to 3 small brain metastases. Metastases in the eloquent cortex, basal ganglia, thalamus, and even the brainstem, a location considered to be surgically inaccessible, can be treated with a relatively low risk [10]. In clinical practice, surgery should be considered for any patient with a single brain metastasis in an accessible location, especially if the tumor is large, the mass effect is significant, and/or obstructive hydrocephalus is present [11]. In our case, SRT was not chosen because the tumor size was 4.5 cm and it was accessible by surgery.

In whole-brain radiation therapy (WBRT) for brain metastases, a total of 30 to 50 Gy, divided in 10–20 fractions is applied, and with a single dose is 1.5 -3 Gy [8, 9]. WBRT alone could be a treatment option for patients with multiple brain metastases or poor performance status patients [11]. WBRT is sometimes supplemented with surgery or SRT to treat micrometastases [8, 9]; however, there is no definitive evidence of overall survival imporvement if WBRT is performed after surgery, instead of SRT. Additionally, the risk of late neurotoxicity after WBRT in long-surviving patients is not negligible. Up to 11% of patients who underwent WBRT experienced neurotoxicity symptoms, including memory loss that progressed to dementia, frontal gait disorders, and urinary incontinence [11].

In our case, the LR of YST, discovered 4 years after operation was localized in a surgically accessible area of the patient’s brain. SRT was not chosen but a metastasectomy was performed in consideration of the tumor size and location. Because tumor resection was successful, chemotherapy or cerebral radiotherapy was not applied due to concerns about chemoresistance and late neurotoxity. There has been no recurrence 24 months post-operation. Thus, surgery is considered an optimal treatment choice of the LR area if surgically accessible.

## Consent

Written informed consent was obtained from the patient for publication of this Case report and any accompanying images. A copy of the written consent is available for review by the Editor of this journal.

## Abbreviations

LR: Late relapse
GCT: Germ cell tumor
YST: Yolk sac tumor
CT: Computed tomography
AFP: Alpha-fetoprotein
LDH: Lactate dehydrogenase
HCG: Human chorionic gonadotrophin
BEP: Bleomycin, etoposide, and cisplatin
EP: Etoposide and cisplatin
MRI: Magnetic resonance imaging
SRT: Stereotactic radiotherapy
WBRT: Whole-brain radiation therapy.
